# Comparative effectiveness of anterior and posterior approaches for interscalene brachial plexus block: A systematic review and meta-analysis

**DOI:** 10.1016/j.bjane.2024.844574

**Published:** 2024-11-17

**Authors:** Luis Eduardo Ciconini, Theodoro Beck, Catreen Abouelsaad, Karandip Bains, Mauren F. Carbonar

**Affiliations:** a SUNY Downstate Health Sciences University, Department of Anesthesiology, New York, USA; b SUNY Downstate Health Sciences University, New York City, USA

**Keywords:** Block, brachial plexus, Brachial plexus anesthesia, Cervical plexus blockade, Nerve blockade, Anesthesia, Regional anesthesia

## Abstract

**Introduction:**

Interscalene Brachial Plexus Blocks (ISBPB) are highly effective forms of anesthesia for surgeries involving the upper arm, shoulder, and neck. Recently, there has been a growing interest in comparing the advantages and limitations of the anterior and posterior approaches.

**Methods:**

This systematic review and meta-analysis aimed to determine whether the anterior or posterior approach to ISBPB offers a clinical advantage regarding complete block rates and time to block completion. We included randomized controlled trials comparing the anterior and posterior techniques for ISBPB while excluding studies with overlapping populations, comparisons of blocks other than interscalene, and articles written in a non-English language.

**Results:**

The search strategy identified 2229 articles, of which six Randomized Controlled Trials (RCTs) met the inclusion criteria for the meta-analysis. A total of 414 patients were included, with 210 patients in the anterior group and 204 in the posterior group. The Odds Ratio (OR) for a complete sensory block between the two techniques did not reach statistical significance (OR = 0.56 [0.20, 1.58], 95% CI, p = 0.27). Similarly, the Standardized Mean Difference (SMD) for the time to complete the block also did not reach statistical significance (SMD: -0.77 [-2.12, 0.59], 95% CI, p = 0.27). Heterogeneity for complete block was not significant (I^2^ = 0%), while procedure time showed high heterogeneity (I^2^ = 97%).

**Conclusion:**

Both techniques have shown effectiveness in providing surgical analgesia. The choice of technique should be determined by the provider's comfort and proficiency, as well as ensuring the highest level of safety for the patient.

## Introduction

Interscalene Brachial Plexus Block (ISBPB) is a highly effective form of anesthesia for surgical procedures on the upper arm, shoulder, and neck. It anesthetizes most of the territory innervated by the brachial plexus, sparing the inferior trunk.^1^, ^2^, ^3^, ^4^, ^5^, ^6^ Initially done using the landmark technique, the ISB technique has changed and, through ultrasound guidance, enhanced precision and accuracy.^7^, ^8^, ^9^, ^10^, ^11^, ^12^

Pioneered by Winnie, the anterior technique for ISB involves injecting local anesthetic between the anterior and middle scalene muscles at the C6 level and has gained popularity.^13^ However, in 1990, Pippa described a posterior approach to the ISBPB, which consisted of placing the needle where the levator scapulae's posterior border meets the trapezius muscle's anterior border and then advancing the needle towards the brachial plexus within the middle scalene muscle. Compared to the anterior approach, Pippa's technique prevents the local anesthetic from rapidly spreading along the anterior scalene muscle.^11^^,^^14^^,^^15^

Despite the extensive use of both techniques, there remains uncertainty regarding their comparative effectiveness and clinical utility. Previous studies show conflicting results, with some suggesting that the posterior approach may offer advantages in terms of block precision and reduced side effects, while others highlight the greater familiarity that physicians have with the anterior approach.^14^^,^^16^^,^^17^ Furthermore, questions remain about the relative safety, cost-effectiveness, and required skill level for each technique, which are critical considerations in clinical practice. However, no consensus has emerged, underscoring the need for a rigorous comparison of these approaches.

To address these gaps in knowledge, we conducted a comprehensive systematic review and meta-analysis of randomized controlled trials to evaluate whether the anterior or posterior approach to ISBPB offers a clear clinical advantage. Specifically, we compared the two techniques in terms of time to finish the block and complete sensory block rate, with the goal of providing evidence-based guidance for anesthesiologists in choosing the most effective approach.

## Methods

### Search strategy

**Figure 1:**
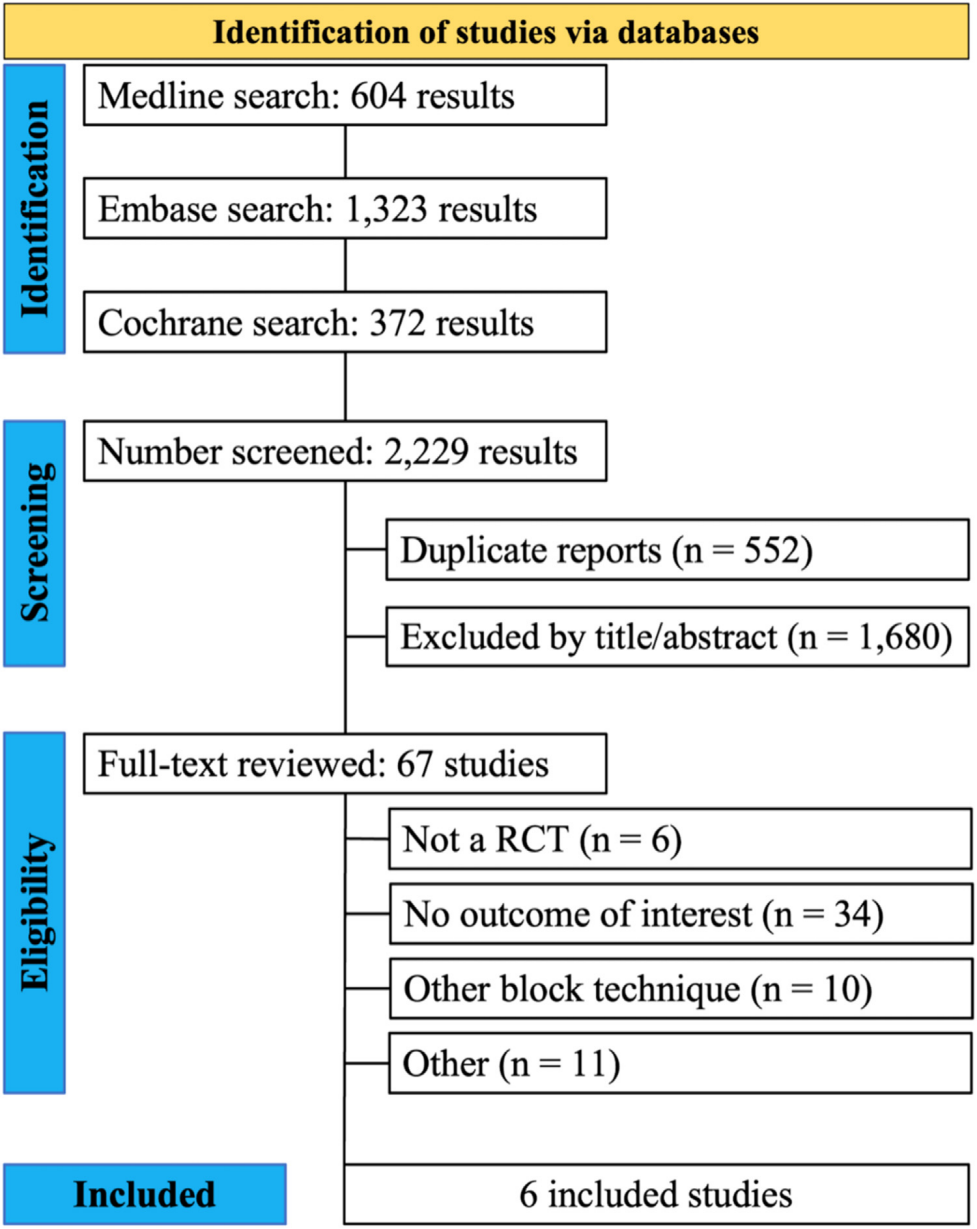
PRISMA flow diagram of study screening and selection.

We conducted a systematic review of the literature from its inception [19,64] to June 2023, using three databases: Medline, Embase, and the Cochrane Library (Figure 1). The search strategy was as follows: “("brachial plexus" OR "scalene" OR "interscalene") AND ("block" OR blockades OR catheter) AND ("lateral" OR "posterior" OR anterolateral OR anterior OR pippa OR winnie).” No filters were applied. The search was supplemented by screening the references of included articles to identify any additional studies.

The systematic review and meta-analysis was performed in accordance with recommendations from the Cochrane Collaboration and the Preferred Reporting Items for Systematic Reviews and Meta-Analysis (PRISMA) statement guidelines^18^ The complete literature search strategy is listed in Table S1 (Supplementary Material).

This meta-analysis was registered on Prospero on October 3, 2023, under the inscription CRD42023469151.^19^ No amendments to information provided at registration or in the protocol were done.

### Eligibility criteria

Two authors independently conducted a literature review. Articles selected for full review by either author were then jointly assessed. We included randomized controlled trials comparing the anterior and posterior interscalene block techniques and their variations. Studies were excluded if they involved overlapping populations, compared non-interscalene blocks, or were published in languages other than English.

### Statistical analysis and data collection

The outcomes of interest were modeled using a random effects model. Using a standardized extraction form, two authors independently extracted data on complete block rate, procedural time, study design, population characteristics, and intervention details.

Standardized mean difference was used to pool continuous outcomes with a 95% Confidence Interval, and Odds Ratio was used to pool categorical outcomes with a 95% Confidence Interval. Heterogeneity was evaluated with the Cochran Q test and I2 statistics; p-values inferior to 0.10, and I2 > 25% were considered significant for heterogeneity. DerSimonian and Laird's random effects model was used in pooled outcomes with high heterogeneity. Review Manager 5·4.1 (Nordic Cochrane Center, The Cochrane Collaboration, Copenhagen, Denmark) was used for statistical analysis. Studies that reported results in terms of median and interquartile intervals had their mean and standard deviations calculated based on these values. Sensitivity analysis was performed for outcomes with high heterogeneity.

### Quality assessment

**Figure 2:**
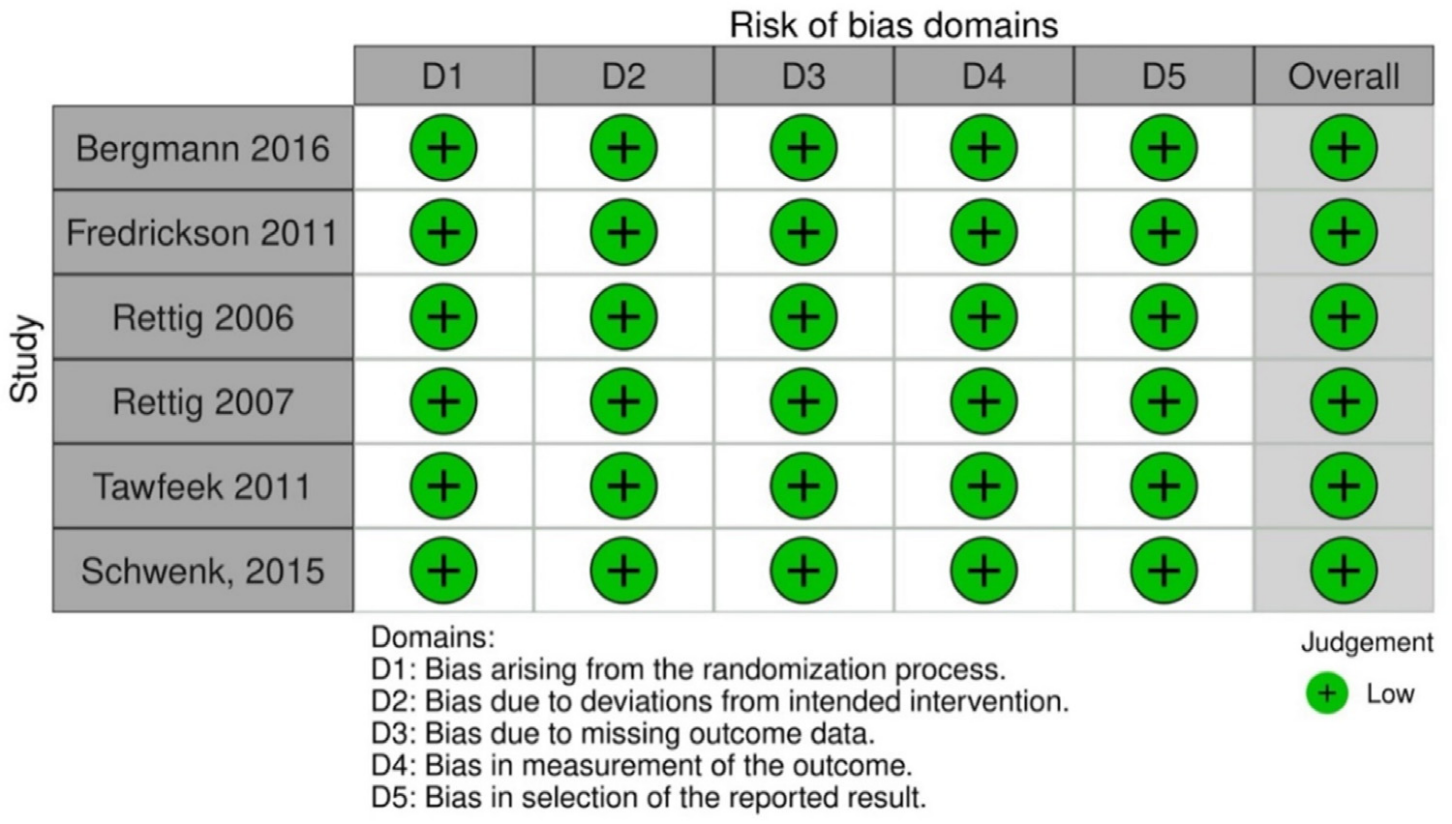
Quality assessment and risk of bias.

The risk of bias was assessed for each included study using the Cochrane Collaboration's Risk of Bias Version 2 (RoB2). Each study was evaluated across five domains: selection bias, performance bias, detection bias, attrition bias, and reporting bias (Figure 2). Bias in individual studies was categorized as high, low, or unclear risk based on predefined questions and flowgrams from each specific domain.

The assessment as performed independently by two reviewers. A spreadsheet was created for visual display of individual results. Disagreements were resolved through consensus or by a third reviewer who acted as a mediator. If consensus was not reached after discussion, the third reviewer would make a final decision.

### Role of funding

There was no funding source for this study. All authors had full access to all the data, and the corresponding author had final responsibility for the decision to submit it for publication. There were no competing interests of any authors.

## Results

A total of 1747 articles were screened through Medline, Cochrane, and Embase databases (Figure 1). Of these, 1680 were excluded based on predetermined criteria, and 67 were read in full. Six randomized controlled trials were included for review and meta-analysis. The sum of patients from all studies was 414, with 210 patients in the anterior group and 204 in the posterior group. The mean age ranged from 38.3 to 63.8 years in the anterior group and from 37.7 to 67.1 years in the posterior group (Table 1).

**Table 1:** Design and characteristics of studies included in the meta-analysis.

Study	Design	Patients^a^	Age^a^^,^^b^	Procedure	Intervention	Location; Period	Population
Bergmann, 2016^11^	RCT	42/42	49.9/48.9	Elective shoulder surgery	ISB	Germany, 2016	ASA I‒II
Fredrickson, 2011^17^	RCT	56/52	48/47	Elective shoulder surgery	ISB	New Zealand, 2009-20010	ASA I‒II
Tawfeek, 2011^31^	RCT	20/20	38.3/ 37.7	Elective shoulder surgery	ISB	Egypt, 20011	ASA I, II, III
Rettig, 2006^16^	RCT	40/40	49.6/46.4	Elective shoulder or upper arm surgery	ISB	Netherlands, 2006	ASA I‒II
Rettig, 2007^44^	RCT	10/10	56/48	Elective shoulder surgery	ISB	Ireland, 2007	ASA I‒II
Schwenk, 2015^45^	RCT	42/40	63.8/67.1	Open shoulder surgery	ISB	United States, 2015	ASA I, II, III

a Anterior Approach (AA) / Posterior Approach (PA).

b Age represented as mean.

Complete block was defined as the lack of need for sedation, adding a block supplement, or converting to general anesthesia due solely to pain in the brachial plexus distribution. Four studies reported complete blocks as an outcome. No study found a statistically significant difference in the number of complete blocks when comparing the two techniques. Studies’ results and outcomes can be seen in Table 2.

**Table 2:** Studies’ outcomes and procedures’ characteristics.

Authors	Primary Outcome	Local Anesthetic	Intraoperative Management	Catheter Infusion	Findings
Bergmann, 2016^11^	Incidence of phrenic nerve block.	15 mL of ropivacaine 1%	Sedation: 0.033 mg.kg^-1^ midazolam, 0.067 μg.kg^-1^ sufentanil, and 0.0133 mg.kg^-1^ propofol.	Non-available.	Both groups experienced significant decreases in respiratory function after an interscalene brachial plexus block, but no significant differences were found.
Fredrickson, 2011^17^	Pain free rates in the recovery room.	20 mL of ropivacaine 0.375% administered preoperatively.	General anesthesia: laryngeal mask airway, desflurane anesthesia, alfentanil 0.25 mg was administered as needed for a respiratory rate greater than 25 breaths per minute.	Ropivacaine 0.2% was administered via elastomeric pump delivering 2 mL.hr^-1^ with patient-controlled boluses of up to 5 mL.hr^-1^.	The anterior group had a statistically significant higher rate of pain relief in the recovery room compared to the posterior group. Rescue tramadol use was higher for the posterior group on the first day after surgery but not on the second day.
Tawfeek, 2011^31^	efficacy and safety of continuous posterior and anterior interscalene brachial plexus blockades.	Lidocaine 2% and Bupivacaine 0.5% (20 mL of both).	Premedication: 3.75–7.5 mg midazolam orally. General anesthesia: 1–2 mcg.kg^-1^ fentanyl, 1.5–2 mg.kg^-1^ propofol, and 0.5 mg.kg^-1^ atracurium. Maintenance: mixture of nitrous oxide (50%–70%) and isoflurane (1%–1.5%) in oxygen, and incremental doses of atracurium.	Infusion of 8‒10 mL.h^-1^ of bupivacaine 0.25% throughout the interscalene catheter was started postoperatively	There was no significant difference regarding the onset of anesthesia in both groups. Block procedure time and catheter placement time were faster in the posterior group.
Rettig, 2006^16^	Clinical efficacy of anterior versus posterior approach of brachial plexus block.	0.5 mL.kg^-1^ of ropivacaine 7.5 mg.mL^-1^	Sedation for the block: alfentanil 0.5 mg and, increment dose mg (0.5) or midazolam 1mg as needed. General anesthesia for surgery: propofol, 2 mg.kg^-1^; fentanyl, 100‒150 mcg for induction and maintained by propofol 4 to 6 mg.kg^-1^.h^-1^ and 70% N_2_O in oxygen.	Non-available.	Anterior and posterior approaches of the brachial plexus were equal and comparable in clinical efficacy for shoulder and upper arm anesthesia.
Rettig, 2007^44^	Concentration of arterial plasma ropivacaine.	Single 3.75 mg.kg^-1^ injection of ropivacaine 7.5 mg.mL^-1^.	Sedation: alfentanil up to 1.0 mg and, if necessary, with midazolam up to 1.0 mg. Opioids were given during surgery as needed.	Non-available.	No statistically significant difference in plasma concentration of ropivacaine was observed between the anterior and posterior groups.
Schwenk, 2015^45^	Visual Analog Scale Pain Scores (VAS) at 24 and 48 hours.	Ropivacaine 0.5% (30 mL).	General anesthesia: propofol 1‒2 mg.kg^-1^, rocuronium, fentanyl 1‒2 mcg.kg^-1^. Maintenance: sevoflurane or desflurane in addition to fentanyl as needed.	Continuous 0.2% ropivacaine at 10 mL.hr^-1^. Bolus of 5 mL of ropivacaine 0.2% was given by the pain team for pain score > 5/10.	No statistical difference in VAS at 24 hours and 48 hours, mean procedure time, and morphine consumption at 24 hours.

**Figure 3:**
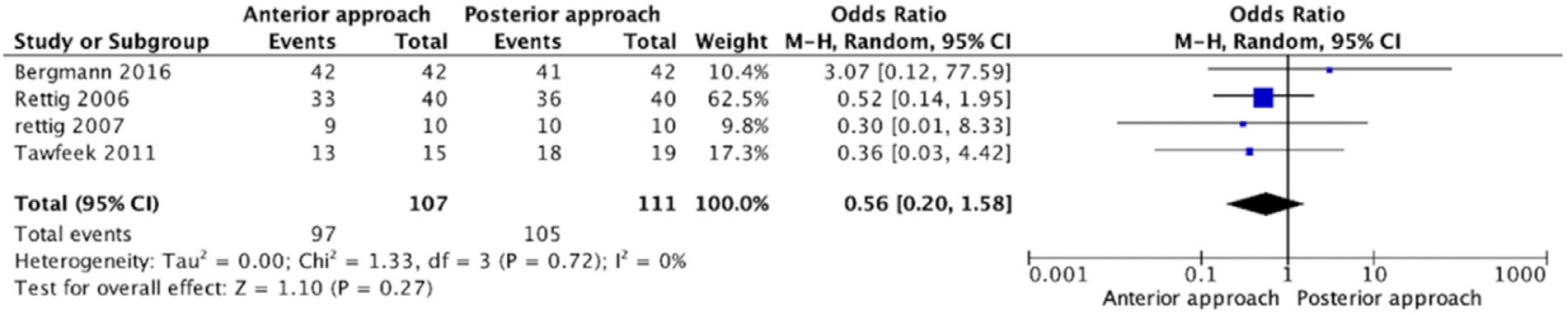
Odds ratio of complete sensory blocks.

In our meta-analysis, the odds ratio for complete blocks between the anterior and posterior approach did not reach statistical significance (OR = 0.56 [0.20, 1.58], 95% CI, p = 0.27), as shown in Figure 3. I^2^ = 0, which is not statistically significant for heterogeneity. The risk of bias was of low concern for the studies included in this outcome.

**Figure 4:**
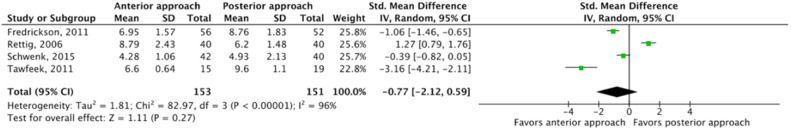
Standardized mean difference of procedure time.

Four studies reported procedure times ranging from 4.28 to 8.79 minutes in the anterior group and 4.93 to 9.6 minutes in the posterior group. No significant difference was observed for this outcome between the two groups (SMD: -0.77 [-2.12, 0.59], 95% CI, p = 0.27), as demonstrated in Figure 4. However, we obtained an I^2^ = 97%, which is statistically significant for heterogeneity. The risk of bias was of low concern for the studies included in this outcome.

Given the substantial heterogeneity in procedure time (I2 = 97%), we performed a sensitivity analysis to explore potential sources of variability. We excluded the study with the fastest time for block completion (Schwenk, 2015) (SMD: -0.87 [-3.05, 1.18], 95% CI, p = 0.38; I2 = 97.66%). This analysis did not significantly alter the overall findings, suggesting that the heterogeneity is likely attributable to variability in practice techniques rather than data outliers.

One potential factor that contributed to the heterogeneity was the variability in operator experience. The included studies did not consistently report the experience level of the practitioners performing the blocks, which could significantly influence procedure time. Additionally, variations in local anesthetic volume may have influenced procedure times.

## Discussion

Regional anesthesia techniques like the Interscalene Brachial Plexus Block (ISBPB) are widely recognized as effective and safe methods for surgical analgesia and postoperative pain management.^5^^,^^20^ These techniques can enable outpatient surgical procedures and reduce hospital admissions for poorly controlled pain.^21^, ^22^, ^23^

The anterior approach for brachial plexus blockade, first described by Winnie in 1970, has been thoroughly studied and is known for its efficacy and relatively high safety profile.^14^^,^^15^^,^^24^^,^^25^ Conversely, the posterior approach, as described by Pippa, has gained popularity for shoulder and upper arm surgeries.^26^ Pippa's technique offers improved visualization of the needle shaft and tip, potentially allowing for more precise needle positioning and reducing the risk of complications.^27^^,^^28^

### Interpretation of results

A total of 1747 articles were screened, with six randomized controlled trials ultimately included in the meta-analysis, encompassing 414 patients (210 in the anterior group and 204 in the posterior group). The meta-analysis found no statistically significant difference between the anterior and posterior approaches regarding the odds ratio for complete blocks. Similarly, the procedure time did not differ significantly between the two groups.

Three studies reported a nonsignificant trend toward greater complete block rate with the anterior approach, while one suggested a nonsignificant advantage for the posterior approach. However, when pooling results from all studies, no significant difference was observed, demonstrating the effectiveness of both techniques. Regarding time to completion, three studies favored the anterior approach, with two reaching statistical significance. In contrast, only one study showed a statistically significant advantage for the posterior approach. Overall, there was no difference in the median time to perform the block when all studies were combined. This variability in results likely reflects differences in operator familiarity with each technique, leading to faster completion times when performing the more familiar method.

Our meta-analysis showed that both the anterior and posterior approaches for Interscalene Brachial Plexus Block (ISBPB) were equally effective in terms of complete block rates and procedure time. These findings have important implications for clinical practice. Since neither approach demonstrated a clear advantage, the choice between the two techniques should primarily be guided by the clinician's expertise, the surgical context, and patient-specific factors. For example, the anterior approach, which is more familiar to most anesthesiologists, may be the preferred technique in most cases. However, the posterior approach might be advantageous in cases where visualization of the needle tip and shaft is crucial, potentially reducing complications related to needle misplacement. Additionally, patient anatomy, co-existing conditions such as obesity, or previous neck surgeries may influence the feasibility of one approach over the other. Importantly, in patients with significant pulmonary compromise (e.g., chronic obstructive pulmonary disease), both techniques carry the risk of phrenic nerve block, requiring careful consideration of patient safety regardless of the technique chosen. Those findings align with current guidelines and best practices in regional anesthesia. Current guidelines do not prefer one approach over another but emphasize the need for safety considerations and an individualized approach.

### Comparison with existing literature

The results of our study were consistent with some previous research but differ from others, highlighting the ongoing debate in the literature about the superiority of different approaches. Previous studies show varying success rates for anterior interscalene non-stimulating catheter techniques, with some achieving 100% success while others report failure rates as high as 20%.^29^^,^^30^ These mixed results have led to debates about the relative merits of the anterior and posterior approaches. There is no clear consensus on which technique is superior in terms of time to completion or success rate.

Comparative studies on postoperative outcomes, such as opioid consumption, procedure time, and patient satisfaction, also yield inconsistent findings. For example, Fredrickson et al. reported that Pippa's technique has more difficulty with catheter threading and longer placement time. In contrast, patients in the anterior group experience higher rates of pain relief and lower opioid consumption within the first 24 hours post-surgery.^17^ However, potential bias is noted due to the operator's greater experience with Winnie's technique. On the other hand, Tawfeek et al. reported faster block procedure times, lower pain scores, and higher patient satisfaction with the posterior approach. It should be noted that this study did not include subcutaneous tunneling in the anterior approach, which may have influenced the results.^31^

Similarly, Fredrickson et al. suggested that the anterior approach may provide better postoperative pain control, with patients requiring less rescue analgesia in the Post-Anesthesia Care Unit (PACU) compared to the posterior approach.^17^ However, our meta-analysis found no significant differences in the number of complete blocks or procedure times between the two techniques. These findings, however, suggest potential advantages of the anterior approach in managing postoperative pain.

Regarding catheter stability, Aoyama et al. did not observe any significant differences between the anterior and posterior approaches concerning catheter tip migration.^32^ Although this outcome was not directly addressed in our meta-analysis, our findings support that both techniques are mechanically equivalent in achieving a complete block, implying that the choice of technique may not significantly affect block success when using catheters.

Rettig et al. did not find any significant differences in block success or patient satisfaction between the approaches.^16^ Overall, most randomized trials, including this meta-analysis, do not favor one technique over the other. Neither approach showed a significant difference regarding complete block rate, procedure time, patient satisfaction, catheter tip migration, phrenic nerve block rate, or local anesthetic plasma concentration. Both techniques are equally effective in achieving a successful block and ensuring patient satisfaction.

### Complications

Despite the long-standing use of Winnie's technique, complications such as subdural anesthesia, subarachnoid injection of local anesthetic, and recurrent laryngeal nerve blockade have been reported.^33^, ^34^, ^35^, ^36^, ^37^ Similarly, Pippa's technique has been associated with complications, including intrathecal and epidural injections, raising safety concerns.^27^^,^^33^^,^^38^^,^^39^

The anterior and posterior approaches risk accidental phrenic nerve blockade, leading to hemidiaphragmatic paresis.^40^, ^41^, ^42^, ^43^ This complication is particularly concerning for patients with severe Chronic Obstructive Pulmonary Disease (COPD). Studies, such as those by Bergmann et al., show no difference between the two approaches regarding the frequency of unintentional phrenic nerve block, suggesting neither technique offers a clear safety advantage.^11^

Catheter dislodgment is another concern with continuous neuraxial and regional anesthesia. The posterior approach is theorized to be more favorable for maintaining the catheter tip position due to its longer insertion length and passage through multiple muscle layers.^24^^,^^25^ However, Aoyama et al. did not find any difference in catheter tip migration between the two approaches in their study of continuous interscalene plexus blocks.^32^

Rettig et al. compared Winnie's and Pippa's approaches and found no significant clinical differences, except for a thoracic epidural block complication observed in one patient with the anterior technique.^16^ Transient symptoms of paresthesia and dysesthesia were reported in both groups, with a higher incidence in the anterior group, though these symptoms generally resolved within 6 to 8 weeks after surgery. In a following study, Rettig et al. analyzed the difference in plasma concentration of local anesthetic following brachial plexus blockade using Winnie's or Pipa's technique. Rettig et al. concluded that both the anterior and posterior techniques yield similar results.^44^ Consequently, as plasma levels of local anesthetics are similar between the two groups, no method, most likely, provides a better safety profile to local anesthetic systemic toxicity, and standard precautions should be taken irrespective of the approach used.

### Limitations

Our study has several limitations that should be considered when interpreting the findings. First, despite including multiple Randomized Controlled Trials (RCTs), the final sample size of 414 patients is relatively small. This limited sample size may restrict the statistical power of our analyses, potentially affecting the generalizability of the results. Larger trials would be needed to confirm these findings and detect more subtle differences between the anterior and posterior approaches.

Second, there is significant heterogeneity in the procedure time analysis (I^2^ = 97%), suggesting variability in operator experience, technique, and study methodologies. This variability could introduce bias, impacting the reliability of our findings. Differences in ultrasound-guided techniques, needle orientation, or local anesthetic volume may have contributed to the variation across studies. Although sensitivity analyses were conducted to mitigate this, the potential for bias remains a concern.

Third, the exclusion of non-English studies introduces the possibility of language bias. It is possible that relevant studies published in other languages were missed, which could limit the comprehensiveness of our review. Similarly, publication bias should also be considered, particularly the non-reporting of negative or inconclusive results. Studies showing no significant differences between techniques may be less likely to be published, skewing the evidence base in favor of either approach.

Moreover, while the included studies were generally classified as having low concerns for bias according to the Cochrane Collaboration tool, significant potential biases were identified in the studies by Fredrickson et al. and Tawfeek et al. These biases, which included variations in operator experience and the use of different adjuncts or techniques (e.g., subcutaneous tunneling), could undermine the strength of their findings. These factors should be accounted for when interpreting the results of this meta-analysis.

Lastly, the generalizability of our findings may be limited by the relatively narrow range of patient populations included in the analyzed RCTs. Most studies focused on elective surgery populations, and the outcomes may not be applicable to patients with more complex medical conditions or those undergoing emergency procedures.

To the best of our knowledge, this meta-analysis represents the only comparative analysis of Pippa's and Winnie's techniques to date. Our study aimed to investigate whether these two techniques led to different outcomes, such as procedure time and complete sensory block. Our findings indicate no significant difference in complete block rate or procedure time between the two techniques. Consequently, contrary to some randomized controlled trials, this meta-analysis demonstrates no obvious superiority of one technique. The observed differences in previous randomized trials can largely be attributed to biases, such as differences in expertise or failure to implement specific steps in one approach.

## Conclusion

Our meta-analysis compared the anterior and posterior approaches for Interscalene Block (ISB), finding no significant differences in speed or success rates between the two techniques. However, our findings suggest that the anterior approach, followed by catheter placement, may offer superior postoperative pain control, and reduced opioid consumption in PACU. Patients with large anterior neck masses, such as those with goiter or thyroid cancer, may benefit more from the posterior approach due to anatomical considerations. Each technique has distinct advantages and risks, so the choice of approach should be tailored to the provider's expertise and the individual patient's clinical scenario. Further research is needed to evaluate long-term outcomes, including complication rates and patient satisfaction.

## Conflicts of interest

The authors declare no conflicts of interest.

## Appendix Supplementary materials

Image, application 1

## Footnotes

Supplementary material associated with this article can be found in the online version at doi:10.1016/j.bjane.2024.844574.
