# Supplementary material for: Comparative effectiveness of anterior and posterior approaches for interscalene brachial plexus block: A systematic review and meta-analysis
Source: Braz J Anesthesiol. 2024 Nov 17;75(1):844574. doi: 10.1016/j.bjane.2024.844574 (PMC11625346; doi:10.1016/j.bjane.2024.844574)
Supplement: Supplementary file 1 [file mmc1.docx]

**BJAN-D-24-00108_Supplementary Material**

**Supplementary Table S1** Search strategy and number of results for each database.

| **Search strategy** | Search terms that resulted in the articles screened: ("brachial plexus" OR "scalene" OR "interscalene") AND ("block" OR blockades OR catheter) AND ("lateral" OR "posterior" OR anterolateral OR anterior OR pippa OR winnie). |
| --- | --- |
| **Database** | **Number of results** |
| MEDLINE | 604 |
| Embase | 1,323 |
| Cochrane | 372 |

The search strategy below was used for all three databases (Medline, Embase, and Cochrane Library). The systematic review retrieved studies from inception [1964] to June 2023. To retrieve the greatest number of articles with possible outcomes of interest, no filter was used.
